# Iron and folic acid consumption and changing social norms: cluster randomized field trial, Odisha, India

**DOI:** 10.2471/BLT.20.278820

**Published:** 2021-08-31

**Authors:** Rajiv N Rimal, Hagere Yilma, Erica Sedlander, Satyanarayan Mohanty, Lipika Patro, Ichhya Pant, Srimant K Khuntia, Minati Swain, Satyaranjan Behera

**Affiliations:** a Department of Health, Behavior, and Society, Johns Hopkins University, 624 N Broadway, Baltimore, MD 21205, United States of America (USA).; b Department of Prevention and Community Health, George Washington University, Washington, DC, USA.; c Department of Family and Community Medicine, University of California San Francisco, San Francisco, USA.; d DCOR Consulting, Bhubaneswar, Odisha, India.; e IPE-Global, New Delhi, India.

## Abstract

**Objective:**

To assess whether improvements in social norms related to iron and folic acid consumption are associated with increased iron and folic acid consumption.

**Methods:**

In a cluster randomized trial in Odisha, India, we implemented an intervention to improve descriptive norms (people’s perceptions about how many other people take iron and folic acid), injunctive norms (social pressures people feel to take iron and folic acid) and collective norms (actual levels of iron and folic acid consumption). We assessed changes in these norms and self-reported iron and folic acid consumption in control and intervention arms after 6 months (September 2019–February 2020). We collected data from control (*n* = 2048) and intervention (*n* = 2060) arms at baseline and follow-up (*n* = 1966 and *n* = 1987, respectively).

**Findings:**

At follow-up, mean scores in self-reported iron and folic acid consumption in the control arm had decreased from 0.39 to 0.31 (21% decrease; not significant). In the intervention arm, mean scores increased from 0.39 to 1.62 (315% increase; *P* < 0.001). The difference between the two arms was statistically significant (*P* < 0.001). Each of the three norms also improved at significantly higher rates in the intervention than in the control arm (*P* < 0.001 for each norm). Changes in descriptive and collective norms (but not injunctive norms) were associated with changes in self-reported iron and folic acid consumption (*P* < 0.001 for both norms).

**Conclusion:**

Our results show that social norms can be improved and that these improvements are associated with positive behavioural changes. A social norms-based approach may help promote iron and folic acid consumption in India.

## Résumé

**Objectif:**

Déterminer si l'évolution des normes sociales en matière de consommation de fer et d'acide folique a permis d'accroître cette consommation.

**Méthodes:**

Dans le cadre d'un essai randomisé en grappes dans l'Odisha, en Inde, nous avons mené une intervention visant à améliorer les normes descriptives (perception des gens vis-à-vis du nombre de personnes prenant du fer et de l'acide folique), injonctives (pression sociale ressentie par les gens, les incitant à prendre du fer et de l'acide folique) et collectives (niveaux de consommation réels pour le fer et l'acide folique). Nous avons évalué les changements affectant ces normes, ainsi que la consommation déclarée de fer et d'acide folique dans les groupes d'intervention et de contrôle après 6 mois (septembre 2019 – février 2020). Nous avons récolté des données dans les groupes de contrôle (*n* = 2048) et d'intervention (*n* = 2060) au début de l'essai et lors du suivi (*n* = 1966 et *n* = 1987, respectivement).

**Résultats:**

Lors du suivi, les scores moyens en termes de consommation déclarée de fer et d'acide folique au sein du groupe de contrôle ont diminué de 0,39 à 0,31 (baisse de 21%; non significative). Au sein du groupe d'intervention, les scores moyens sont passés de 0,39 à 1,62 (hausse de 315%; *P* < 0,001). La différence entre les deux groupes s'est avérée statistiquement significative (*P* < 0,001). Les trois normes ont également connu une amélioration nettement plus rapide dans le groupe d'intervention que dans le groupe de contrôle (*P* < 0,001 pour chacune d'entre elles). Les changements apportés aux normes descriptives et collectives (mais pas injonctives) ont entraîné des changements au niveau de la consommation déclarée de fer et d'acide folique (*P* < 0,001 pour chaque norme).

**Conclusion:**

Nos résultats montrent que les normes sociales peuvent évoluer, et qu'une telle évolution est liée à des modifications comportementales positives. L'adoption d'une approche fondée sur ces normes pourrait contribuer à promouvoir la consommation de fer et d'acide folique en Inde.

## Resumen

**Objetivo:**

Evaluar si las mejoras en las normas sociales relacionadas con el consumo de hierro y ácido fólico se asocian a un mayor consumo de hierro y ácido fólico.

**Métodos:**

En un ensayo aleatorizado por grupos en Odisha, India, se implementó una intervención para mejorar las normas descriptivas (las percepciones de las personas sobre cuántas otras personas consumen hierro y ácido fólico), las normas por mandato (las presiones sociales que las personas sienten para que tomen hierro y ácido fólico) y las normas colectivas (los niveles reales de consumo de hierro y ácido fólico). Se evaluaron los cambios en estas normas y el consumo de hierro y ácido fólico informado por los participantes en los grupos de control y de intervención después de 6 meses (septiembre de 2019 a febrero de 2020). Se recopilaron datos de los grupos de control (*n*= 2048) y de intervención (*n*= 2060) al inicio y en el seguimiento (*n*= 1966 y *n*= 1987, respectivamente).

**Resultados:**

En el seguimiento, las puntuaciones medias en el consumo de hierro y ácido fólico informado por los participantes en el grupo de control disminuyeron de 0,39 a 0,31 (disminución del 21 %; no significativa). En el grupo de intervención, las puntuaciones medias aumentaron de 0,39 a 1,62 (315 % de aumento; *P* < 0,001). La diferencia entre los dos grupos fue estadísticamente significativa (*P* < 0,001). Cada una de las tres normas también mejoró a tasas muy superiores en el grupo de intervención que en el de control (*P* < 0,001 para cada norma). Los cambios en las normas descriptivas y colectivas (pero no en las normas por mandato) se asociaron con los cambios en el consumo de hierro y ácido fólico informado por los participantes (*P* < 0,001 para ambas normas).

**Conclusión:**

Los resultados de este ensayo demuestran que las normas sociales se pueden mejorar y que estas mejoras se asocian a cambios positivos del comportamiento. Un enfoque basado en las normas sociales puede ayudar a promover el consumo de hierro y de ácido fólico en la India.

## ملخص

**الغرض:**

تقييم ما إذا كانت التحسينات في الثوابت الاجتماعية المتعلقة باستهلاك الحديد وحمض الفوليك مرتبطة بزيادة استهلاك الحديد وحمض الفوليك.

ا **لطريقة **في تجربة عشوائية مجمعة في أوديشا بالهند، قمنا بتنفيذ تدخل لتحسين الثوابت الوصفية (تصورات الأشخاص حول عدد الأشخاص الآخرين الذين يتناولون الحديد وحمض الفوليك)، والمعايير التحذيرية (الضغوط الاجتماعية التي يشعر بها الأشخاص لتناول الحديد وحمض الفوليك)، والثوابت الجماعية (المستويات الفعلية لاستهلاك الحديد وحمض الفوليك). قمنا بتقييم التغييرات في هذه الثوابت، واستهلاك الحديد وحمض الفوليك الذي أبلغ عنه الأشخاص ذاتيًا في أذرع التحكم والتدخل بعد 6 أشهر (سبتمبر/أيلول 2019 إلى فبراير/شباط 2020). قمنا بجمع البيانات من أذرع التحكم (العدد = 2048)، والتدخل (العدد = 2060) عند خط الأساس والمتابعة (العدد = 1966 والعدد = 1987، على التوالي).

**النتائج:**

أثناء المتابعة، انخفض متوسط الدرجات في استهلاك الحديد وحمض الفوليك الذي أبلغ عنه الأشخاص ذاتيًا في ذراع التحكم من 0.39 إلى 0.31 (انخفاض بنسبة 21%؛ ليس ملموسًا). في ذراع التدخل، زاد متوسط الدرجات من 0.39 إلى 1.62 (زيادة بنسبة 315%؛ نسبة الاحتمال أقل من 0.001). كان الفرق بين الذراعين ذي دلالة إحصائية (نسبة الاحتمال أقل من 0.001). تحسن كل من الثوابت الثلاثة أيضًا بمعدلات أعلى بشكل ملموس في ذراع التدخل عنه في ذراع التحكم (نسبة الاحتمال أقل من 0.001 لكل من الثوابت). ارتبطت التغييرات في الثوابت الوصفية والجماعية (ولكن ليس الثوابت التحذيرية) بالتغيرات في استهلاك الحديد وحمض الفوليك الذي أبلغ عنه الأشخاص ذاتيًا (نسبة الاحتمال أقل من 0.001 لكلا الثابتين).

**الاستنتاج:**

تظهر نتائجنا أنه يمكن تحسين الثوابت الاجتماعية، وأن هذه التحسينات مرتبطة بالتغيرات السلوكية الإيجابية. قد يساعد الأسلوب القائم على الثوابت الاجتماعية في الارتقاء باستهلاك الحديد وحمض الفوليك في الهند. ****

## 摘要

**目的:**

旨在评估与铁和叶酸消耗相关的社会规范的改善是否与铁和叶酸消耗量的增加有关。

**方法:**

在印度奥里萨邦的一项集群随机试验中，我们实施了一项干预措施以改善描述性规范（人们对有多少人服用铁和叶酸的看法）、指令性规范（人们对服用铁和叶酸感受到的社会压力）和群体性规范（铁和叶酸消耗的实际水平）。我们对这些规范的变化进行了评估，并对 6 个月后（2019 年 9 月至 2020 年 2 月）对照组和干预组中铁和叶酸的消耗量进行了自我报告。我们从对照组（n = 2048）和干预组（n = 2060）收集了基线数据和后续数据（分别为 n = 1966 和 n = 1987）。

**结果:**

在后续跟踪中，自我报告显示对照组中铁和叶酸消耗量的平均分值从 0.39 下降到 0.31（下降了 21%；不显著）。干预组中的平均分值从 0.39 增加到 1.63（增加了 315%；*P* < 0.001）两组之间具有统计上的显著差异（P < 0.001）。三个规范中的每一个规范在干预组的改善都显著高于对照组（每个规范 *P* < 0.001）。描述性和集体性规范（不包括指令性规范）的变化均与自我报告中的铁和叶酸消耗量的变化有关（两种规范的 P < 0.001）。

**结论:**

我们的结果表明社会规范可以得到改善，并且这些改善与积极的行为改变有关。基于社会规范采取的方法可能有助于促进印度地区铁和叶酸的消耗量。

## Резюме

**Цель:**

Оценить, связаны ли улучшения социальных норм, относящихся к потреблению железа и фолиевой кислоты, с увеличением потребления железа и фолиевой кислоты.

**Методы:**

В кластерном рандомизированном исследовании, проводимом в штате Одиша, Индия, авторы осуществили мероприятие, направленное на улучшение дескриптивных норм (представления людей о том, какое количество человек принимают железо и фолиевую кислоту), предписательных норм (социальное давление, которое люди испытывают, чтобы принимать железо и фолиевую кислоту) и коллективных норм (фактические уровни потребления железа и фолиевой кислоты). Была проведена оценка изменения этих норм и самоотчетности о потреблении железа и фолиевой кислоты в группе контроля и экспериментальной группе после 6 месяцев (сентябрь 2019 г. — февраль 2020 г.). Авторы собрали данные группы контроля (*n* = 2048) и экспериментальной группы (*n* = 2060) на исходном уровне и в период последующего наблюдения (*n* = 1966 и *n* = 1987 соответственно).

**Результаты:**

В период последующего наблюдения средний балл по самоотчетности о потреблении железа и фолиевой кислоты в группе контроля снизился с 0,39 до 0,31 (снижение на 21%; несущественное). В экспериментальной группе средний балл увеличился с 0,39 до 1,62 (увеличение на 315%; *P* < 0,001). Разница между двумя группами была статистически значимой (*P* < 0,001). Также каждая из трех норм улучшалась со значительно более высокими показателями в экспериментальной группе, чем в группе контроля (*P* < 0,001 для каждой нормы). Изменения дескриптивных и коллективных норм (но не предписательных норм) были связаны с изменениями самоотчетности о потреблении железа и фолиевой кислоты (*P* < 0,001 для обеих норм).

**Вывод:**

Наши результаты показывают, что социальные нормы можно улучшить и что эти улучшения связаны с положительными изменениями поведения. Подход, основанный на социальных нормах, может способствовать увеличению потребления железа и фолиевой кислоты в Индии.

## Introduction

More than 1.6 billion people worldwide and more than half of Indian women of reproductive age have iron deficiency anaemia.^1^^,^^2^ Anaemia leads to fatigue, preterm delivery risk and maternal mortality.^3^^,^^4^

The Indian government has invested substantial resources to increase iron and folic acid consumption to reduce anaemia.^5^^–^^7^ India’s National Nutritional Anaemia Control Programme promotes iron-rich foods and offers free iron and folic acid supplements to pregnant and breastfeeding women.^6^ The National Iron Plus Initiative provides iron and folic acid tablets to adolescents in schools and free supplements to pregnant women.^7^ Nevertheless, anaemia rates in India remain high.^2^^,^^5^ Therefore, the question remains as to how we can increase uptake of iron and folic acid supplements to reduce the anaemia burden in India. Social norms may be an approach to achieve this goal.

Social norm theories assert that people’s behaviours are guided, to some extent, by the behaviours of others in their midst and their own understanding of the expectations of other people.^8^ The influence of social context on behaviour goes beyond anaemia and research shows that three types of social norms can influence behaviours: descriptive, injunctive and collective norms (Box 1).^8^^–^^13^ Individuals engage in a behaviour when they believe other people are also engaging in this behaviour (i.e. descriptive norms) and that other people approve of that behaviour (injunctive norms).^14^^–^^16^ While descriptive and injunctive norms refer to perceptions about the behaviour of other people, collective norms refer to the true prevalence of a behaviour within a community.^17^ This norm, and not just the perception of norms, has been shown to influence behaviours.^9^^,^^18^^,^^19^

Box 1 **Definitions of norms used in the paper**

**Descriptive norms**

People’s perceptions about the prevalence of a behaviour.^8^ When people believe most other people engage in a particular behaviour, descriptive norms are said to be high. Conversely, descriptive norms are low when people believe a particular behaviour is uncommon.

**Injunctive norms**

Social pressure people feel to conform. When people believe that other people expect them to behave in a certain way, injunctive norms are said to be high. So-called peer pressure is another term used to describe injunctive norms.^8^

**Collective norms**

The actual prevalence of a behaviour.^9^ Whereas descriptive and injunctive norms relate to people’s perceptions and beliefs, collective norms describe the extent to which a particular behaviour is common in an area. High collective norms signify a high proportion of people in an area engaging in the particular behaviour.

In this study, we investigated the influence of descriptive, injunctive and collective norms on iron and folic acid consumption through an intervention conducted by the Reduction in Anaemia through Normative Innovations Project in Odisha, in the east of India. The primary outcome of this field trial is reduction in anaemia as measured through haemoglobin level. We used data collected at the midpoint of the intervention to analyse a secondary outcome – the change in people’s iron consumption behaviours. Specifically, we considered whether a field experiment could improve social norms and, if so, whether those improvements would lead to improvements in iron and folic acid consumption.

## Methods

Beginning in January of 2019, we first conducted a 6-month mixed-method formative research project in two out of eight blocks (Kishorenagar and Athmalik) in Angul district in Odisha, the site of our work.^20^^,^^21^ Blocks are administrative units similar to a postal code. The findings of this research (Box 2) informed the overall project intervention, which was delivered from the beginning of September 2019 until the end of February 2020. The intervention to improve iron and folic acid consumption was based on the theory of normative social behaviour,^22^ which includes both descriptive and injunctive norms.

Box 2 **Results from the formative assessments that informed the intervention, Odisha, India**

Formative assessment findings^19^ highlighted the need to incorporate gender-based approaches, given that women of reproductive age in Odisha were at considerable disadvantage because they: eat last in the home, after most of the food (and nutrients) have been consumed; do not prioritize their own health over that of their children, husbands and mothers-in-law; have lower autonomy to seek self-care; and have less say in financial decision-making in the home to allocate family resources for their own health. Findings also showed that women of reproductive age did not view fatigue as necessarily a problem, seeing it as a non-medical issue.^20^ Rather, their own identity as a woman and as a mother or wife was connected with the physical tasks they had to perform, which would lead to feeling tired. This finding led us to design the Reduction in Anaemia through Normative Innovations Project intervention by adopting a gender perspective that targeted not only women of reproductive age (our focal audience) themselves, but also their social networks (e.g. husbands and mothers-in-law) and community leaders. We also saw the need to provide education to communicate that fatigue was part of anaemia and that anaemia could be treated by taking iron and folic acid tablets.

### Study design

In this cluster randomized controlled trial, we grouped two to four adjacent villages (total = 239) into 89 clusters. Using a random number generator we randomly assigned clusters to either the intervention (50 clusters with 130 villages) or the control (39 clusters with 109 villages) arm. These groups comprised the two arms for intervention delivery.

**Fig. 1:**
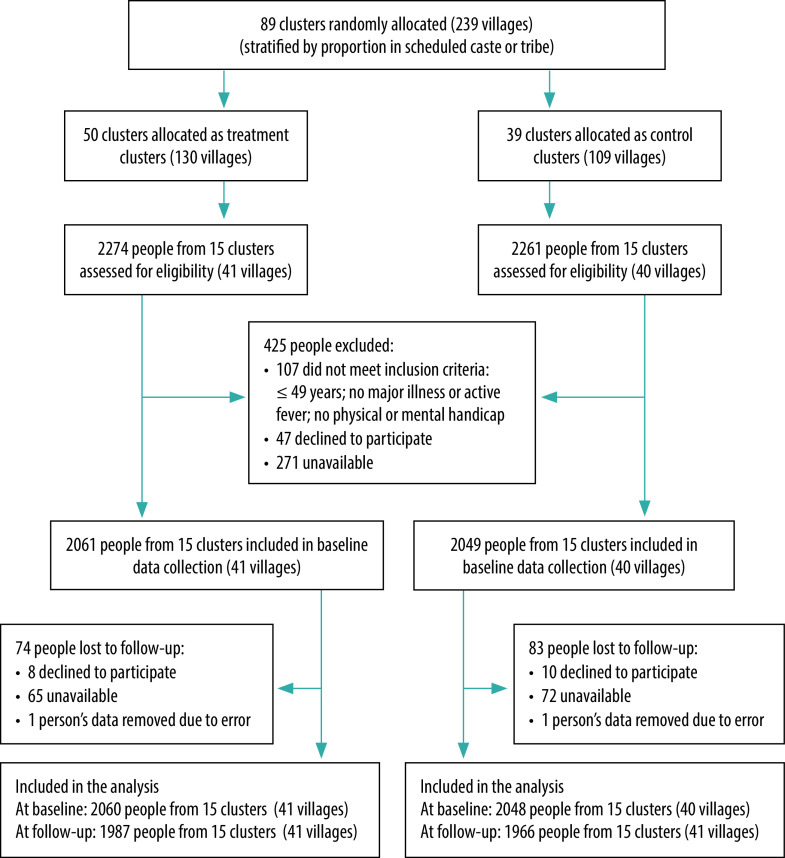
Flowchart of inclusion of participants in the cluster randomized trial to increase iron and folic acid consumption, at baseline and at follow-up, Odisha, India, September 2019–February 2020

We stratified villages in each arm by the proportion of the population belonging to a scheduled caste or tribe (officially designated groups of people in India), as they represent socially marginalized groups. We then used a random number generator to select clusters with the highest, medium and lowest proportion of scheduled caste or tribe. This selection resulted in 15 clusters within each arm (41 villages in the intervention arm and 40 in the control arm). These clusters comprised the two arms for data collection. More details about the study design are described elsewhere^23^ and shown in Fig. 1.

We blinded the data collection team to the status of any given home (whether intervention or control and whether providing data or not), although in the course of data collection, team members could have surmised the status of the home. Apart from the statistician allocating the randomization, no one else was informed about the intervention or control status of any given village.

### Intervention

Box 3 shows the components of the intervention we used to improve social norms and, ultimately, to increase iron and folic acid consumption.

Box 3 **Intervention components of the Reduction in Anaemia through Normative Innovations Project, Odisha, India**

**Educational modules (4Ts)**

The educational component of the intervention had four components:

(i) Teaching: educating community facilitators about the social, behavioural, biomedical and normative aspects of anaemia;

(ii) Training: coaching the facilitators on how to convey this information to our target audience;

(iii) Tuning: adapting the overall intervention approach as new information becomes available; and

(iv) Talking: promoting discussions to spread campaign messages.

We developed 10 modules (each 1 hour long) on topics related to anaemia control, including iron and folic acid supplementation, diet diversity, social norms, malaria, water and sanitary hygiene, and deworming. We adapted these modules, developed through our ongoing work in various parts of India, for the local context in the local language. Each module included interactive activities and games structured around prevailing norms identified during the formative research.

**Haemoglobin testing**

In each of the 130 intervention villages, we tested haemoglobin levels of 15 women every month, using HemoCue metres that provided instant digital results. To convey the test results in a meaningful way, we designed blood-shaped cards of different colours indicating anaemia severity: green for anaemia free (≥12 g/dL, or 120 g/L), yellow for mild anaemia (11–11.9 g/dL, or 110–119 g/L), orange for moderate anaemia (8–10.9 g/dL, or 80–120 g/L), and red for severe anaemia (< 8 g/dL, or < 80 g/L). After the test, women received the appropriate card, together with relevant behavioural suggestions and recommendations for consuming iron-rich foods and iron and folic acid tablets.

We shared test results with participants in three forms of feedback: ipsative (comparing the woman’s current haemoglobin reading with her readings in the past); normative (comparing the woman’s current haemoglobin result with those of other people in her community); and aspirational (comparing the woman’s current haemoglobin reading with healthy haemoglobin levels). All women received normative and aspirational feedback; only women who had had previous haemoglobin tests within the project received ipsative feedback. We also tabulated the readings for each community, and we presented the three feedback methods in aggregate (e.g. the community’s progress over time, the community average compared with the averages of two neighbouring communities, and the community current average compared with an anaemia-free reading for the community). We shared test results (aggregated to protect privacy) with other key stakeholders, including policy-makers.

**Communication videos**

During the formative research, we collected local stories to develop short videos. Each video was 3–4 minutes long and was shot locally using local residents as actors. The videos highlighted the key messages of the programme (including modelling positive social norms around iron and folic acid consumption) and addressed barriers identified during the formative research. We showed the videos to small groups on tablet computers and individual viewers also watched them on smartphones. We presented a new video each month; at the same time, we showed the previous videos to women who had missed watching them earlier.

**Supply monitoring**

We initially conceived the Reduction in Anaemia through Normative Innovations Project as a demand-side intervention, and we did not consider supply-side barriers in designing the campaign. The Government of India’s existing programmes are supposed to provide the necessary medication doses to the states, which then distribute them down to their communities. Over the course of our intervention, we began using unused iron and folic acid supplies and foresaw potential shortages in the future. As a result we had to start supply-side intervention activities, including advocacy efforts with the local administration officials and training frontline workers to monitor and proactively act when the iron and folic acid supply appeared to be falling.

To bring about change in descriptive norms – people’s perceptions about the prevalence of a behaviour in their social networks – the intervention materials communicated that more and more women were beginning to consume iron and folic acid to reduce anaemia. To bring about change in injunctive norms – the pressure people feel to conform – the intervention materials depicted the level of support that women of reproductive age could expect to receive from others in their communities, including their mothers-in-law, husbands, friends and government officials. 

While videos communicated this information, we also made use of social norms by having the target audience for each video (women of reproductive age, husbands and other men in the community, adolescent girls and government officials) watch not only the specific video targeted to them but also the videos that targeted other groups. For example, videos that targeted adolescent girls were also shown to mothers-in-law. The idea was to promote the notion that all the other people in the communities were now supporting women of reproductive age and promoting iron and folic acid consumption.

Community facilitators delivered the intervention, many of whom came from the communities they served. These facilitators met monthly to review progress, share what they had learnt, and incorporate new findings. To improve the adaptability and sustainability of the approach, we developed an advanced monitoring system (called real-time performance monitoring for knowledge) through which intervention components and feedback from recipients were uploaded after the completion of each activity. The system then updated a dashboard that allowed us to gauge progress and share the data with the government and other stakeholders.

### Data collection

An all-female team collected data at baseline and again at 6-month follow-up. We hired another team to ensure data quality, and to observe and evaluate the performance of each data collector several times throughout the survey period. All variables examined in this paper are described in the data repository.^24^

#### Iron and folic acid consumption

At both baseline and follow-up, women self-reported how many iron and folic acid tablets they had consumed in the past week.

#### Descriptive norms

Based on previous studies,^22^^,^^25^^,^^26^ we assessed descriptive norms through three questions on the participants’ perceptions of the frequency with which other community members consumed iron and folic acid tablets. The scores for the responses to each of the three questions ranged from 0 (none) to 4 (all). We averaged the responses to the three questions into a scale at baseline (Cronbach α = 0.45) and follow-up (Cronbach α = 0.63).

#### Injunctive norms

We measured injunctive norms, the extent to which women believed that important people in their lives^27^^,^^28^ expected them to consume iron and folic acid tablets,^26^ by asking how many other people supported or disapproved of their taking iron and folic acid tablets. In addition, we asked participants about perceived support from their mothers-in-law (or most mothers-in-law, if unmarried) and husbands (or most husbands, if unmarried). We scored responses on a five-point Likert scale ranging from strongly disagree to strongly agree, and averaged the scores into a scale at baseline (Cronbach α = 0.71) and follow-up (Cronbach α = 0.77).

#### Collective norms

Collective norms assess the actual behaviour in one’s social network or environment. We calculated the non-self-mean of behaviour (i.e. the relative prevalence of the behaviour after taking out the value of the person whose score was being calculated) within a geographical unit.^9^ We calculated the average number of iron and folic acid tablets consumed in each village (minus the respondent’s score to reduce biased covariance with the outcome) at baseline and follow-up.

#### Participants’ characteristics

We also controlled for some demographic and anthropometric variables. For example, body mass index is associated with anaemia,^29^ and we obtained height and weight measurements of the participants. Other factors associated with anaemia in India are age, education, parity and caste membership,^30^ and we assessed these variables as well.

### Statistical analysis

We tested the proposition that social norms had improved as a result of the intervention by using difference-in-difference analyses,^27^ comparing changes from baseline to follow-up across intervention and control arms. We used hierarchical multilinear regression equations to test the idea that self-reported iron and folic acid consumption was greater in the intervention than the control groups, and that improvement in norms were themselves associated with improvements in self-reported behaviours. We further asked whether improvements in the norms would have a differential effect on self-reported iron and folic acid consumption in the intervention arm compared with the control arm. We created interaction terms between the intervention and each norm and tested their influence on the outcome variable. To avoid multicollinearity, each interaction term was tested separately, one at a time.

### Ethical considerations

The Indian Council for Medical Research’s Health Ministry Screening Committee approved the study. We obtained ethical approval for this study from the Institutional Review Board at George Washington University, USA (the recipient of the funding) and the Institutional Review Board of Sigma Science and Research, New Delhi, India. The trial is registered with the Clinical Trial Registry of India (CTRI/2018/10/016186).

Before data collection, we obtained written informed consent in accordance with the Sigma Institutional Review Board protocol from each participant. For participants younger than 18 years we obtained their assent supplemented by informed consent from their parent or guardian.

## Results

Of the 4110 participants at baseline, we re-interviewed 3955 (96.2%) at 6-month follow-up. We excluded an additional two respondents from the analysis because they were noted as being potentially misidentified from the baseline sample. Differences between those participants retained in the follow-up sample and those who were lost or declined to participate are shown in Table 1. Retained women were significantly younger and less educated, and more likely to be scheduled tribe members and mothers compared with women who were lost at follow-up.

**Table 1:** Characteristics of women retained at follow-up and women lost to follow-up in the cluster randomized trial to increase iron and folic acid consumption, Odisha, India, September 2019–February 2020

Variable	No. (%)		*P*
	Retained (*n* = 3953)	Lost to follow-up (*n* = 157)	
**Age, years**			< 0.001
15–19	454 (11.5)	39 (24.8)	
20–24	712 (18.0)	51 (32.5)	
25–29	780 (19.7)	27 (17.2)	
30–39	1268 (32.1)	23 (14.6)	
40–49	739 (18.7)	17 (10.8)	
**Education, years of schooling**			< 0.001
None	729 (18.4)	21 (13.4)	
1–6	1102 (27.9)	25 (15.9)	
7–10	1626 (41.1)	68 (43.3)	
11–12	370 (9.4)	24 (15.3)	
≥ 13	126 (3.2)	19 (12.1)	
**Tribal and caste group**			< 0.01
Scheduled tribe member	1118 (28.3)	35 (22.3)	
Scheduled caste member	544 (13.8)	38 (24.2)	
Other	2291 (58.0)	84 (53.5)	
**No. of children**			< 0.001
None	898 (22.7)	79 (50.3)	
1	811 (20.5)	39 (24.8)	
2	1374 (34.8)	24 (15.3)	
≥ 3	870 (22.0)	15 (9.6)	

Notes: Lost to follow-up were women who declined to continue, were excluded or dropped from the analysis. We calculated *P* values using *χ^2^* test.

Table 2 gives the characteristics of the sample in the control and intervention arms at baseline. The control arm included significantly fewer scheduled tribe members than the intervention arm. Other differences were not significant.

**Table 2:** Characteristics of participants in the cluster randomized trial to increase iron and folic acid consumption, Odisha, India, September 2019–February 2020

Variable	No. (%)		*P*
	Control arm (*n* = 2048)	Intervention arm (*n* = 2060)	
**Age, years**			NS
15–19	228 (11.6)	191 (9.7)	
20–24	366 (18.6)	354 (17.9)	
25–29	388 (19.7)	395 (20.0)	
30–39	621 (31.6)	644 (32.6)	
40–49	362 (18.4)	393 (19.9)	
Missing data	83 (4.2)	83 (4.2)	
**Education, years of schooling**			NS
None	349 (17.8)	375 (18.9)	
1–6	565 (28.7)	537 (27.0)	
7–10	799 (40.6)	831 (41.8)	
11–12	185 (9.4)	172 (8.7)	
≥ 13	68 (3.5)	72 (3.6)	
Missing data	82 (4.2)	73 (3.7)	
**Tribal and caste group**			< 0.001
Scheduled tribe member	1203 (58.7)	1267 (61.5)	
Scheduled caste member	224 (10.9)	320 (15.5)	
Other	621 (30.3)	473 (23.0)	
**No. of children**			NS
None	430 (21.9)	412 (20.7)	
1	422 (21.5)	427 (21.5)	
2	700 (35.6)	733 (36.9)	
≥ 3	414 (21.1)	415 (20.9)	
Missing data	82 (4.2)	73 (3.7)	

NS: not significant.

Note: We calculated *P* values using *χ^2^* test.

The difference-in-differences data are shown in detail in the data repository.^24^ From baseline to follow-up, descriptive norms improved significantly in both the control arm (*t*(1966) = 8.86, *P* < 0.001) and the intervention arm (*t*(1986) = 53.78, *P* < 0.001; Table 3). Improvement in the intervention arm was significantly greater than in the control arm (*t*(3951) = 33.00, *P* < 0.001).

**Table 3:** Mean scores in key variables at baseline and follow-up in control and intervention arms, the cluster randomized trial to increase iron and folic acid consumption in Odisha, India, September 2019–February 2020

Variable	Control arm				Intervention arm				*P* ^a^
	Mean (SD)		*P*		Mean (SD)		*P*		
	Baseline (*n* = 2048)	Follow-up (*n* = 1966)			Baseline (*n* = 2060)	Follow-up (*n* = 1987)			
Descriptive norms	1.08 (0.62)	1.23 (0.62)	< 0.001		1.07 (0.60)	2.06 (0.67)	< 0.001		< 0.001
Injunctive norms	2.66 (0.74)	2.92 (0.75)	< 0.001		2.55 (0.74)	3.60 (0.59)	< 0.001		< 0.001
Collective norms	0.39 (0.32)	0.30 (0.24)	< 0.001		0.39 (0.26)	1.57 (0.87)	< 0.001		< 0.001
Iron and folic acid consumption	0.39 (1.90)	0.31 (1.57)	NS		0.39 (1.92)	1.62 (3.17)	< 0.001		< 0.001

NS: not significant; SD: standard deviation.

^a^ Compares differences in key variables from baseline to follow-up in the control arm with differences in the same variables in the intervention arm.

Note: For all variables, higher scores signify greater iron and folic acid consumption (and supportive norms for this consumption).

Injunctive norms also improved significantly from baseline to follow-up in the control arm (*t*(1965) = 12.29, *P* < 0.001) and the intervention arm (*t*(1986) = 54.69, *P* < 0.001). Improvement in the intervention arm was significantly greater than in the control arm (*t*(3951) = 27.30, *P* < 0.001).

Collective norms in the control arm declined significantly (*t*(1965) = 11.48, *P* < 0.001), but improved in the intervention arm (*t*(1986) = 58.54, *P* < 0.001). The improvement in the intervention arm was significantly greater than in the control arm (*t*(3951) = 58.60, *P* < 0.001).

From baseline to follow-up, mean scores in self-reported iron and folic acid consumption in the control arm decreased slightly from 0.39 to 0.31 (21% decrease; not significant). However, in the intervention arm, mean scores in self-reported iron and folic acid consumption increased significantly from 0.39 to 1.62 (315% increase; *P* < 0.001; Table 3). This difference in improvement between the intervention and control arms was statistically significant (*t*(3951) = 14.04, *P* < 0.001).

In the multivariable analyses of the differences between control and intervention groups (Table 4), we first controlled for self-reported baseline iron and folic acid consumption, which accounted for less than 1% of the variance (*β* = 0.06, *P* < 0.001). In the second model assessing demographic characteristics and body mass index, body mass index was negatively associated with women’s iron and folic acid consumption, while being pregnant was positively associated with iron and folic acid consumption. Parity, education and tribal or caste membership were not associated with iron and folic acid consumption. Overall, this model explained 9.6% of the variance. Controlling for baseline levels, self-reported iron and folic acid consumption at follow-up was significantly greater in the intervention arm than the control arm (*β* = 0.28, *P* < 0.001), which explained 7.6% of the variance.

**Table 4:** Factors associated with iron and folic acid consumption at follow-up: multivariable regression analyses, Odisha, India, September 2019–February 2020

Independent variable	*r* (*P*)^a^	Variables up to the given model included			All main-effect variables included		Difference in *R*^2^
		*β* (SE)^b^	*P*		*β* (SE)^c^	*P*	
**Model 1: Baseline iron and folic acid consumption**	0.11 (< 0.001)	0.06 (0.03)	< 0.001		0.12 (0.02)	< 0.001	0.004 (< 0.001)
**Model 2: Demographic and anthropometric characteristics**							0.096 (< 0.001)
Baseline body mass index	−0.07 (< 0.001)	−0.06 (0.01)	< 0.001		−0.07 (0.01)	< 0.001	
Number of children	−0.02 (NS)	0.02 (0.04)	NS		0.01 (0.03)	NS	
Pregnant at time of data collection	0.33 (< 0.001)	0.30 (0.20)	< 0.001		0.33 (0.19)	< 0.001	
Education	−0.01 (NS)	−0.03 (0.01)	NS		−0.03 (0.01)	NS	
Scheduled tribal or caste member	0.05 (0.01)	0.02 (0.08)	NS		0.02 (0.08)	NS	
**Model 3: Intervention effect (intervention)^b^**	0.25 (< 0.001)	0.28 (0.07)	< 0.001		0.08 (0.11)	< 0.001	0.076 (< 0.001)
**Model 4: Iron and folic acid consumption × change in:^c^**							0.024 (< 0.001)
Descriptive norms	0.18 (< 0.001)	0.05 (0.05)	< 0.01		0.05 (0.05)	< 0.01	
Injunctive norms	0.15 (< 0.001)	0.03 (0.04)	NS		0.04 (0.04)	*<* 0.05	
Collective norms	0.29 (< 0.001)	0.19 (0.05)	< 0.001		0.20 (0.05)	< 0.001	
**Model 5A: Intervention × change in descriptive norms^d^**	0.22 (< 0.001)	0.07 (0.09)	*<* 0.05		0.06 (0.09)	*<* 0.05	0.001 ( *<* 0.05)
**Model 5B: Intervention × change in injunctive norms^d^**	0.20 (< 0.001)	0.05 (0.08)	NS		0.04 (0.08)	NS	0.001 (NS)
**Model 5C: Intervention × change in collective norms^d^**	0.30 (< 0.001)	0.24 (0.16)	< 0.001		0.22 (0.16)	< 0.001	0.003 (< 0.001)
**Total R^2^**							0.204 (< 0.001)

*β*: standardized regression coefficient; NS: not significant; *r*: Pearson correlation coefficient; *R*^2^: explained variance; SE: standard error.

^a^ Pearson correlation between the independent variable and outcome (follow-up iron and folic acid consumption).

^b^ Assignment to intervention (coded as 1) or control (0).

^c^ Improvement in norms from baseline to follow-up (computed as the difference).

^d^ To avoid multicollinearity, only one interaction term was included in the equation for any given model.

Note: For all variables, higher scores signify greater iron and folic acid consumption (and supportive norms for this consumption).

The fourth model tested the proposition that changes in norms would predict changes in self-reported iron and folic acid consumption (Table 4), and showed that changes in descriptive and collective norms were associated with changes in self-reported iron and folic acid consumption (*β* = 0.18, *P* < 0.001 and *β* = 0.29, *P* < 0.001, respectively). However, there was no association for injunctive norms. These factors explained 2.4% of the variance.

With regard to interactions between the intervention and each norm, we found significant interaction between intervention and changes in descriptive and collective norms, although their effects were small. The interaction term between the intervention and change in injunctive norms was not significant. In the control arm, the relationship between changes in descriptive or collective norms and self-reported iron and folic acid consumption was not significant.

## Discussion

The results of our study support the hypothesis that the Reduction in Anaemia through Normative Innovations Project improved descriptive, injunctive and collective norms associated with self-reported iron and folic acid consumption. Improvement in these norms, in turn, affected self-reported iron and folic acid consumption behaviours.

The strength of the bivariate and multivariable associations between baseline and follow-up self-reported iron and folic acid consumption were rather weak (although statistically significant), indicating that other factors, beyond participants’ habitual behaviours, must have accounted for their self-reported consumption at follow-up. Indeed, our multivariable model indicated that social norms and the intervention itself accounted for only about 10% of the variance. In addition, a significant predictor of self-reported iron and folic acid consumption at follow-up was pregnancy status – pregnant women were more likely to report taking iron and folic acid compared with women who were not pregnant. This finding reflects the Indian government’s priority to promote iron and folic acid consumption during pregnancy. In Odisha, pregnant women linked with the health system are integrated into the antenatal continuum of care, through which they receive iron and folic acid tablets. Frontline health workers monitor antenatal consumption, but not postnatal consumption, when women are on their own. The situation among non-pregnant women is different. Even though India’s national anaemia-free priority specifically spells out reducing anaemia among all women of reproductive age, non-pregnant women currently are not being served (despite calls to do so), thus leaving a substantial gap in coverage.^31^

These norms-related findings highlight that perceptions matter: when people believe other people are engaging in a behaviour and perceive pressures to conform, they are more likely to adopt this behaviour. Therefore, interventions to address anaemia need to communicate information about the consumption behaviours of other people to their target audience, highlighting the improving environment. We suspect that through the monthly haemoglobin testing sessions we ran in the communities, this information about improvements in the community was passed on to other women, i.e. that more and more women were taking iron and folic acid which was improving their health. The primary outcome of the overall intervention, that is, assessment of haemoglobin level as an indicator of anaemia, will be assessed at the end of the project and is not reported in this paper.

A significant benefit of norms-based approaches is their sustainability. Descriptive norms in particular exert their influence because of individuals’ motivations to do the right thing, which they deduce by observing and internalizing the behaviours of people close to them as so-called social proof.^32^ These motivations continue to exert their influence even when others are no longer present. Findings from our study indicate that, although how norms play out in a community is culturally bound and contextual, their considerable influence on human behaviour is well established.^15^^,^^33^^,^^34^ This finding points to the generalizability of our study findings, but the extent to which this approach is reproducible and scalable remains to be seen.

The main limitation of this study is that our outcome of interest, self-reported iron and folic acid consumption, is a self-reported behaviour, which could be subject to social desirability bias. Residents in the intervention arm may have known the project team was in their village conducting various activities to promote iron consumption. When interviewers subsequently asked about their behaviours, they exaggerated their consumption. While we cannot rule this bias out, it does not completely explain the outcome for two reasons. First, data collectors were not part of the intervention team (and were blinded to the intervention or control status of their interview sites). As a result, we created some separation between the intervention and data collection teams. Second, not only did we observe greater consumption in the intervention arm than the control arm, but we also observed greater changes in norms and significant associations between normative changes and behavioural changes. Social desirability bias alone could not explain these outcomes.

Another limitation relates to the scope and generalizability of the findings. The study was conducted in one district in Odisha and, even then, in only two blocks out of eight. The extent to which our study area is representative of the larger part of the district or the state remains to be seen. We do not know, for example, whether similar findings would emerge in parts of the state more heavily populated by tribal populations. While this issue relates to external validity, the internal validity of our study remains fairly robust.

We did not assess women’s income to gauge and control for their economic and financial status. To some extent, the randomized design overcomes this drawback. Furthermore, women in our study sites (at least in theory) can access iron and folic acid tablets for free from the government; in reality, few of them do so, particularly when they are not pregnant.

Despite these limitations, the study has several strengths. First, the study has a good internal validity design, which allows for causal inferences between the intervention and iron and folic acid consumption. Second, the underlying mechanism of change is also theoretically sound as it conforms with predictions from theories of social norms.^7^^,^^17^^,^^26^ Finally, for a health condition such as anaemia – the prevalence of which is still high,^1^ particularly in India –^2^^,^^5^ our findings indicate that a social norms-based approach may provide the first step in bringing about change, at least behaviourally, in promoting iron and folic acid consumption.

Our paper shows that social norms can serve as important mechanisms through which behaviours are enacted and changed. However, this finding has to be understood with regard to the characteristics of the behaviour itself. In the case of our study, the behaviour appears both simple (take one iron and folic acid pill every day) and under the control of the individuals. What we found, however, is that the behavioural drivers are multifaceted. Women will not adhere to the recommendations to take iron and folic acid pills if they do not believe anaemia is a serious health issue; if they do not prioritize their own health above that of others they care for; and if they are not supported by other people in their communities. Some of these drivers relate to perception. We found that both descriptive and injunctive norms (both of which are assessed as beliefs) were instrumental in promoting self-reported iron and folic acid consumption; women who believed many others were consuming iron and folic acid tablets and that their social networks supported them in doing so were themselves more likely to comply. Other drivers were not based on perceptions but on the actual behaviour of other people. We found that collective norms (which capture the extent to which women come from communities in which iron and folic acid consumption is high or low) were significant predictors of self-reported iron and folic acid consumption.

In India, because pregnant women come in contact with the health system more often than non-pregnant women, the health system is much more geared to supply tablets to pregnant women. This may explain why self-reported iron and folic acid consumption at baseline among non-pregnant women was so low. While the intervention of the Reduction in Anaemia through Normative Innovations Project appears to be improving iron and folic acid consumption among both pregnant and non-pregnant women, more clearly needs to be done by the health system to ensure non-pregnant women comply with the recommended dose.

This rural setting of our study shares similar challenges to those faced by other middle-income countries where women’s reduced levels of empowerment, decision-making and autonomy combine to suppress progress on their health and well-being (as has been the case for anaemia in India). We anticipate that a social norms-based approach, of the type adopted in this project, can shift the responsibility for change from women themselves to their larger community, regardless of whether they are in low- or middle-income countries.
